# Successful Long-Term Survival Following Thyroid Storm Induced by Radioactive Iodine Therapy in a Dog with Thyroid Carcinoma

**DOI:** 10.3390/vetsci12121164

**Published:** 2025-12-06

**Authors:** Dasom Son, Byeong-Teck Kang, Younju Kim, Taesik Yun, Hakhyun Kim, Yeon Chae

**Affiliations:** 1 Laboratory of Veterinary Internal Medicine, College of Veterinary Medicine, Chungbuk National University, Cheongju 28644, Republic of Korea; dasom4293@gmail.com (D.S.); kangbt@chungbuk.ac.kr (B.-T.K.); fermium@chungbuk.ac.kr (T.Y.); kimh@chungbuk.ac.kr (H.K.); 2 Korea Animal Medical Center, Cheongju 28651, Republic of Korea; vet-kim5362@hanmail.net

**Keywords:** canine, thyroid carcinoma, thyroid storm, radioactive iodine therapy

## Abstract

An 8-year-old castrated male Pomeranian with a non-resectable functional thyroid carcinoma and concurrent myxomatous mitral valve disease was referred for radioactive-iodine therapy. Due to clinical thyrotoxicosis at referral and concurrent cardiac disease, the radioiodine dose was selected conservatively at the lower end of the reported therapeutic range. Despite a conservative radioactive iodine dose, the dog developed acute thyrotoxic decompensation consistent with thyroid storm (manifesting as anxiety, diarrhea, hyperthermia, hypersalivation, and marked tachycardia) within hours of treatment. Propranolol and butorphanol administration led to rapid clinical stabilization. Before the second radioactive iodine therapy, methimazole and propranolol were used for subsequent management, effectively controlling thyrotoxicosis risk and enabling a higher radioiodine dose. Serum thyroxine normalized within 1 month after the second treatment, and the dog maintained complete clinical remission thereafter. Radioactive iodine therapy served as definitive therapy to prevent recurrent life-threatening thyrotoxicosis, resulting in a euthyroid state and long-term survival. This case describes the first documented case of a dog with thyroid carcinoma developing probable thyroid storm associated with radioiodine treatment and subsequently achieving a favorable prognosis.

## Simple Summary

Thyroid storm represents an acute, life-threatening exacerbation of thyrotoxicosis that leads to systemic decompensation. The condition is well recognized in humans, although it appears rare in dogs and cats, with only a few suspected cases reported in veterinary medicine. This case describes a dog with thyroid carcinoma that developed probable thyroid storm following radioactive iodine therapy. Successful management of thyrotoxicosis with propranolol, butorphanol, and methimazole enabled a second radioiodine treatment. The favorable long-term outcome emphasizes the importance of individualized protocols to optimize safety and therapeutic efficacy.

## 1. Introduction

Thyroid storm represents the most severe manifestation of thyrotoxicosis, where excessive thyroid hormone activity triggers acute systemic decompensation [1,2]. Due to rapid progression and multisystem involvement, thyroid storm is considered a medical emergency requiring immediate intervention [3]. In humans, reported mortality for thyroid storm ranges from approximately 80–100% without treatment to 10–50% even with intensive care [3,4]. Diagnosis relies on clinical findings, including hyperthermia, tachyarrhythmias, neurologic abnormalities, and gastrointestinal or hepatic dysfunction. Owing to the non-specific nature of these signs, standardized scoring systems, including the Burch–Wartofsky Point Scale and the Japanese Thyroid Association criteria, are used in human medicine to guide diagnosis [2,5]. In veterinary medicine, thyroid storm appears to be extremely rare, with only a few suspected cases reported in dogs and cats [6,7,8]. The absence of validated veterinary criteria makes diagnosis particularly challenging. Such rarity and diagnostic uncertainty suggest the importance of recognizing probable cases and contribute to the ongoing debate regarding the true occurrence of thyroid storm in animals [9,10].

Radioactive iodine therapy (RAIT) has become an important alternative for managing canine thyroid tumors, particularly in invasive or non-resectable cases, by selectively targeting iodine-avid tissue, including metastases. However, RAIT has been reported to precipitate thyroid storm, potentially through antithyroid drug withdrawal, radiation-induced release of thyroid hormone from damaged follicles, or direct radioiodine effects [11,12,13,14]. After thyroid storm, RAIT can still serve as a definitive treatment to prevent future episodes of life-threatening thyrotoxicosis, although such occurrences are exceedingly rare and a direct association remains uncertain [15,16].

This report describes the first documented case of a dog with thyroid carcinoma that developed probable thyroid storm associated with RAIT and subsequently achieved successful long-term survival. The outcome demonstrates that stabilizing thyrotoxicosis enables subsequent RAIT and suggests that individualized protocols could optimize safety and efficacy in dogs with functional thyroid carcinoma.

## 2. Case Presentation

**Figure 1:**
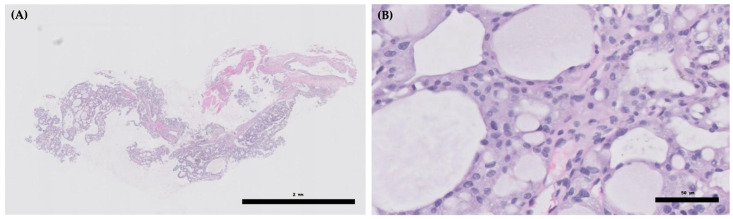
Histopathology of an incisional biopsy specimen from the cervical mass. (**A**) Low magnification (hematoxylin and eosin; scale bar: 2 mm) shows follicular structures within a poorly demarcated mass; (**B**) High magnification (hematoxylin and eosin; scale bar: 50 μm) demonstrates follicular epithelial cells with pleomorphism and colloid-like eosinophilic material within glandular lumina.

**Figure 2:**
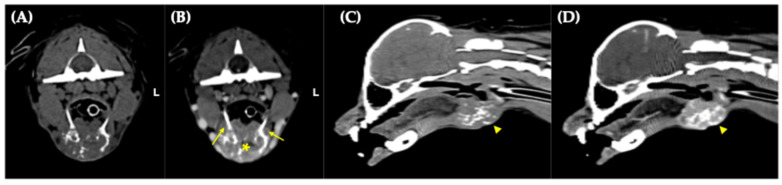
Computed tomography of the cervical mass. (**A**,**B**) Transverse images, pre-contrast (**A**) and post-contrast (**B**), show osteolysis involving the thyrohyoid bone (arrows) and the basihyoid bone (asterisk) with neoplastic invasion; L, left; (**C**,**D**) Sagittal images, pre-contrast (**C**) and post-contrast (**D**), show the craniocaudal extent of the lesion and associated destruction of the hyoid apparatus (arrowheads).

An 8-year-old castrated male Pomeranian presented with a 7-month history of a progressively enlarging cervical mass accompanied by generalized alopecia, progressive weight loss, polyuria, polydipsia, and polyphagia. Histopathological analysis of an incisional biopsy of the cervical mass showed features consistent with a follicular type of thyroid adenocarcinoma (Figure 1A,B). Computed tomography (CT) revealed a 2.8 × 1.7 × 3.2 cm mass with osteolysis of the hyoid bone due to neoplastic invasion (Figure 2A–D). The tumor had infiltrated the thyroid cartilage and extended into the oral cavity beneath the epiglottis, closely abutting the hyoid venous arch and mildly compressing both lingual veins. The dog had been concurrently managed for myxomatous mitral valve disease (MMVD, stage B2) with pimobendan (0.25 mg/kg orally every 12 h), enalapril (0.5 mg/kg orally every 12 h), and furosemide (0.7 mg/kg orally every 12 h) due to concern for impending congestive heart failure, based on echocardiographic evidence of elevated left atrial pressure load. Based on these findings, complete surgical resection was considered unfeasible, and the dog was subsequently referred for RAIT.

**Figure 3:**
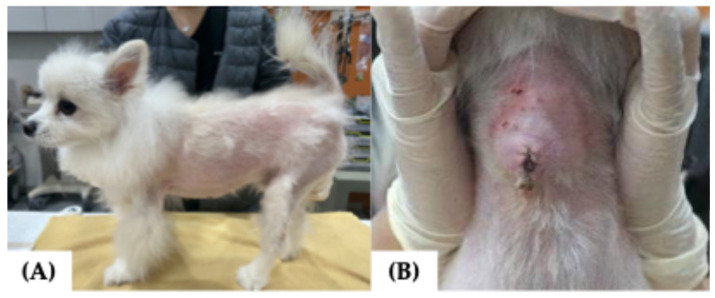
General appearance at referral in a dog presenting with a cervical mass. (**A**) Generalized alopecia and poor body condition were evident; (**B**) a firm cervical mass was visible on inspection.

**Figure 4:**
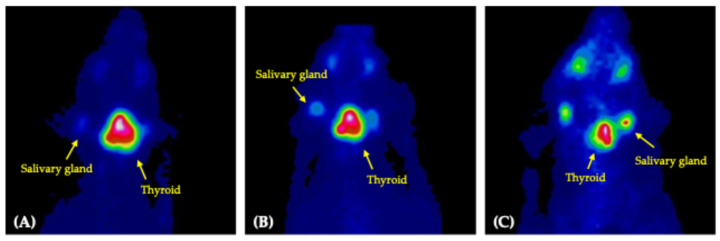
Thyroid scintigraphy with technetium-99m pertechnetate during the treatment course (dorsoventral view). The color display pattern of the rainbow is used in this planar scintigraphy image. (**A**) At referral, marked radionuclide uptake was present in the cervical mass; (**B**) Three months after the first radioactive iodine therapy (RAIT), a hyperthyroid state persisted; (**C**) Eight months after the second RAIT, a euthyroid state was achieved.

At the time of referral, the dog exhibited clinical signs consistent with thyrotoxicosis, including anxiety, tachycardia (222 beats per minute [bpm]), tachypnea (>80 per minute), diarrhea (fecal score 6/7 on 7-point scale), and hypersalivation [2,5,17]. Physical examination revealed a body temperature of 39.0 °C, generalized symmetric alopecia with a poor hair coat, a body weight of 3.08 kg, and a body condition score (BCS) of 3/9, with a firm, approximately 4 cm ventral cervical mass (Figure 3A,B). The serum total thyroxine (tT4) concentration was elevated at 14.9 µg/dL (reference interval [RI], 1.0–4.0 µg/dL), and the thyroid-stimulating hormone (TSH) concentration was <0.030 ng/mL (RI, 0.05–0.42 ng/mL) (Table 1). Thyroid scintigraphy was performed to evaluate the functional status and iodine uptake potential of the tumor and to assess for ectopic thyroid tissue or metastasis. Following intravenous injection of 129.5 MBq of technetium pertechnetate (Tc-99m; New Korea Industrial, Seoul, Republic of Korea) via a cephalic vein, images were acquired 45 min post-injection using a dedicated breast-specific gamma camera (Dilon 6800 Gamma Camera; Dilon Technologies, Newport News, VA, USA) with a low-energy, high-resolution, 15-degree slant parallel-hole collimator. Three standard views (dorsoventral, right lateral, and left lateral) were obtained to assess the cervical region and thoracic inlet. Scintigraphy revealed a 3.2 × 3.2 cm mass with increased Tc-99m uptake (percent thyroidal uptake of 99mTc-pertechnetate [TcTU], 56.6%; thyroid-to-salivary ratio [TSR], 8.09), indicating high iodine uptake potential and suitability for RAIT (Figure 4A). No ectopic thyroid tissue or evidence of metastasis was detected. Based on the World Health Organization clinical staging system, the tumor was classified as T2bN0M0 (clinical stage II) [18]. To achieve pre-treatment stabilization, methimazole (5 mg/dog orally every 12 h; Methimazole^®^, Bukwang Pharmaceutical, Seoul, Republic of Korea) was administered for 1 week, followed by a 3-day withdrawal period before RAIT. At the time of withdrawal, the serum tT4 concentration was 12.1 µg/dL (RI, 1.0–4.0 µg/dL). Upon admission for RAIT, laboratory evaluation revealed mild leukocytosis with neutrophilia, along with elevated liver enzymes, lactate, and C-reactive protein levels (Table 1). Despite these findings, no contraindications for RAIT were identified, and treatment was initiated as planned.

Radioactive iodine (I-131; Thyrokitty, Korea Atomic Energy Research Institute, Daejeon, Republic of Korea) was administered subcutaneously at a dose of 242.831 MBq (78.44 MBq/kg). The dosage was selected considering concurrent MMVD as a potential precipitating factor and the thyrotoxic signs observed at presentation. Approximately 2 h after RAIT, the dog developed acute signs of thyroid storm, including anxiety, sudden aggression, jugular vein pulsation, panting, tachycardia (240 bpm), hyperthermia (39.6 °C), nausea, diarrhea (fecal score 7/7), and hypersalivation. Based on human clinical criteria, these signs corresponded to a Burch–Wartofsky Point Scale score of 60 (Table 2; highly suggestive of thyroid storm [≥45]) and would be classified as suspected thyroid storm according to Akamizu’s diagnostic criteria. The administration of propranolol (0.1 mg/kg orally every 8 h; Indenol^®^, Myungmoon Pharm, Seoul, Republic of Korea) and intravenous butorphanol (0.2 mg/kg as needed; Butophan Injection^®^, Myungmoon Pharm, Seoul, Republic of Korea) achieved clinical stabilization within 4 h. No additional medications or intravenous fluids were required during the subsequent 4-day hospitalization. During this period, all signs resolved as thyrotoxicosis improved, with the exception of diarrhea, which improved to a fecal score of 5/7 by discharge. The measured dose rate at 1 m was 4.8 μSv/h, which was below the established release criterion of 5 μSv/h, and the dog was consequently discharged [19]. Propranolol was continued at discharge to prevent recurrence of thyrotoxicosis and was prescribed twice daily for the owner’s convenience.

At the 1-month follow-up after RAIT, the cervical mass had slightly decreased in size to 2.98 × 1.73 cm on physical examination, with mildly improved yet persistent tachycardia (180 bpm). Although clinical signs showed improvement, the serum tT4 concentration remained elevated at 11.3 µg/dL, prompting the reinitiation of methimazole therapy (5 mg/dog orally every 12 h). Propranolol (0.1 mg/kg orally every 12 h) was continued due to the risk of recurrent thyroid storm and maintained throughout the treatment period. During subsequent follow-up, tT4 concentrations remained elevated despite methimazole dose escalation (increased by 25% at 2-week intervals, from 5.0 to 6.25 and then 7.8 mg/dog orally every 12 h), leading to a second RAIT 3 months after the initial treatment.

Before the second RAIT, methimazole was discontinued for 3 days to prevent thyrotoxicosis, and propranolol (0.1 mg/kg orally every 12 h) was continued. Laboratory evaluation demonstrated mild leukocytosis with neutrophilia, along with elevated liver enzymes, lactate, and C-reactive protein levels, similar to findings before the first treatment (Table 1). Scintigraphy revealed a 3.0 × 2.8 cm mass with increased Tc-99m uptake (TcTU, 45.1%; TSR, 5.2), consistent with sustained functional activity (Figure 4B). The serum tT4 concentration remained elevated at 13.4 µg/dL (RI, 1.0–4.0 µg/dL). Given the thyroid storm that followed the first RAIT, propranolol (previously prescribed every 12 h for the owner’s convenience) was increased to every 8 h during hospitalization, and intravenous dexamethasone (0.01 mg/kg; Jeil Dexamethasone Injection^®^, Jeil Pharm, Seoul, Republic of Korea) was administered before the radioiodine injection. The second RAIT was administered at 386.21 MBq (111 MBq/kg). Within minutes of injection, signs consistent with recurrent thyrotoxicosis, including tachypnea, transient inappetence, aggression, hypersalivation, and diarrhea (fecal score 7/7) developed, and intravenous butorphanol (0.2 mg/kg, as needed) achieved clinical stabilization within 4 h.

At discharge on day 5 post-treatment, the serum tT4 concentration had decreased to 6.49 µg/dL, and methimazole was not prescribed. However, feces remained loose (fecal score 5/7), and stress-induced transient episodes of tachypnea, dyspnea, and cyanosis were observed; therefore, propranolol (0.1 mg/kg orally every 8 h) was continued. At the 1-month follow-up after the second RAIT, the serum tT4 concentration had further decreased to 3.01 µg/dL (RI, 1.0–4.0 µg/dL), accompanied by clinical improvements, including weight gain and resolution of tachycardia and tachypnea. Diarrhea had also improved but not completely resolved, with the fecal score decreasing to 3–4/7 at this time. At that visit, the heart rate, respiratory rate, and body weight were 162 bpm, 36 breaths per minute, and 3.88 kg (BCS 5/9), respectively; propranolol was discontinued.

**Figure 5:**
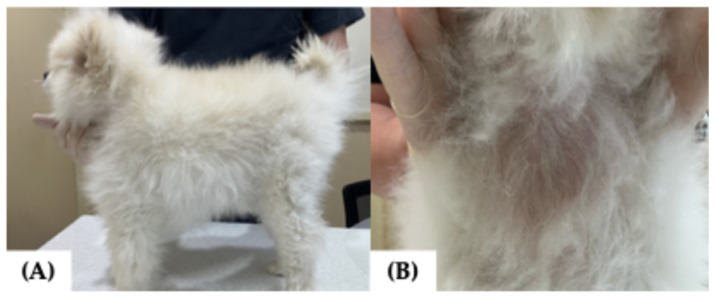
General appearance in a dog at 8 months after the second radioactive iodine therapy (RAIT). (**A**) Body condition improved, and alopecia had resolved; (**B**) the cervical mass was no longer palpable.

Eight months following the second RAIT, physical examination demonstrated normalization of clinical status, with a body weight of 4.3 kg, a BCS of 6/9, and complete resolution of alopecia (Figure 5A,B). Fecal consistency was normal (fecal score 2/7), and no diarrhea was reported. Serum chemistry, complete blood count, and electrolyte profiles were within the RI, except for mild elevations in alkaline phosphatase (302 U/L; RI, 29–97 IU/L) and blood urea nitrogen (31.4 mg/dL; RI, 7–25 mg/dL). The serum tT4 concentration was 1.75 µg/dL (RI, 1.0–4.0 µg/dL), and TSH was 0.052 ng/mL (RI, 0.05–0.42 ng/mL) (Table 1). Thyroid scintigraphy demonstrated normalization of uptake values, with TcTU and TSR of 1.6% and 1.01, respectively, while the residual mass measured 2.8 × 2.3 cm, indicating clinical resolution of the disease (Figure 4C).

## 3. Discussion

This report described the first documented case of a dog with non-resectable thyroid carcinoma that developed probable thyroid storm following RAIT, a rare but potentially life-threatening complication [21]. Prompt recognition and stabilization of the suspected thyroid storm, along with control of thyrotoxic signs, enabled a second RAIT and resulted in sustained euthyroid and long-term survival [3,7,22]. This case suggests that individualized management strategies could minimize adverse events, including thyrotoxicosis, and optimize clinical outcomes in dogs undergoing RAIT.

Functional thyroid carcinoma requires special attention because hormone excess can precipitate cardiovascular complications, including tachyarrhythmias, hypertension, and, in rare cases, thyroid storm. Previous reports in dogs have described only a few suspected cases of thyroid storm associated with functional thyroid carcinoma [6,7]. In humans, a paradoxical thyroid storm can occur after RAIT, potentially triggered by antithyroid drug withdrawal, hormone release from radiation-damaged follicles, or the direct effects of radioiodine [11,12,13,14]. Nevertheless, withdrawal of antithyroid drugs is routinely recommended before RAIT because thionamides reduce thyroidal radioiodine uptake and organification, which can diminish the therapeutic efficacy of radioiodine [23,24]. When thyroidal uptake is suppressed, a larger fraction of the administered activity is distributed to non-target tissues, increasing whole-body and extra-thyroidal radiation exposure and potentially raising the risk of adverse effects [25]. In this dog, methimazole was withdrawn for only 3 days before RAIT as a compromise to allow sufficient thyroidal uptake while minimizing the period of uncontrolled thyrotoxicosis and the risk of another storm, consistent with reports of 2–3 day withdrawal protocols in high-risk human patients [26]. In addition, dose selection required careful consideration because of markedly elevated T4 levels, thyrotoxicosis-consistent signs at presentation, and concurrent MMVD as a precipitating factor for thyroid storm [2,27]. Therefore, the initial dose was selected conservatively as 78.4 MBq/kg, at the lower end of the reported therapeutic range (74–148 MBq/kg) for RAIT in dogs with thyroid carcinoma [28,29,30].

During the first RAIT, the dog showed a clinical presentation consistent with thyroid storm according to both the Burch–Wartofsky Point Scale and Akamizu’s diagnostic criteria [2,5]. Although these scoring systems have been validated for humans, they have been adopted in several canine and feline cases as a practical aid to clinical decision-making under conditions of diagnostic uncertainty [6,8,22]. The scoring was modified by aligning the fever and tachycardia cutoffs with the Veterinary Cooperative Oncology Group—Common Terminology Criteria for Adverse Events definitions [20], and the recalculated Burch–Wartofsky score was 60, exceeding the ≥45 threshold considered highly suggestive of thyroid storm. Akamizu’s diagnostic criteria also require thyrotoxicosis with elevated free T4 or free triiodothyronine (T3), which limits their applicability in veterinary settings [8]. In this case, free T4 was not measured; therefore, despite fulfillment of the other criteria, the episode was classified as “suspected” rather than “definite” thyroid storm. In veterinary emergency practice, rapid free T4 testing remains rarely feasible, requiring clinicians to depend on prior history or previously obtained thyroid profiles, which limits the immediate applicability of these diagnostic criteria.

In human thyroid storm, beta (β)-blockers are recommended for heart rate control and attenuation of adrenergic manifestations, whereas benzodiazepines are commonly used for agitation and anxiety to reduce sympathetic outflow and facilitate care [27,31]. Propranolol is widely used because it controls tachyarrhythmias through β-adrenergic blockade and inhibits peripheral conversion of T4 to T3 [1,7,9,32]. In this case, propranolol administration achieved prompt rate control and clinical stabilization, consistent with both symptomatic relief and physiological benefit. Additionally, butorphanol was used as a short-acting sedative and anxiolytic agent that reduced sympathetic drive, facilitated handling and oxygenation, and likely contributed to hemodynamic stabilization during the acute thyrotoxicosis phase. This therapeutic approach appropriately adapted human treatment guidelines to the canine case, aligned with clinical indications, and facilitated rapid stabilization.

Given the patient’s ongoing requirement for methimazole dose escalation, definitive therapy, including RAIT, remained indicated [3,27]. Retreatment after several months has also been reported, typically within a 3- to 7-month interval, allowing sufficient time to evaluate treatment response and reduce the likelihood of impaired uptake. Consequently, a second RAIT was administered 3 months later to address persistent hyperthyroidism while minimizing the risk of thyroid stunning [28,29,33,34]. For the second RAIT, pre-treatment management included methimazole and propranolol, with dexamethasone administered before radioiodine to further reduce storm risk. Corticosteroids can help prevent relative adrenal insufficiency associated with the hypermetabolic state of thyroid storm and relieve thyrotoxicosis by inhibiting thyroid hormone synthesis and peripheral conversion of T4 to T3 [27]. Effective control of thyrotoxicosis resulted in fewer and milder storm-related signs during the second RAIT, which permitted a higher dose of 111 MBq/kg than the first treatment. The second course achieved normalization of thyroid hormone concentrations, resolution of clinical signs, and long-term survival, supporting the value of individualized dosing strategies in dogs with functional thyroid carcinoma.

## 4. Conclusions

This case documented the occurrence of probable thyroid storm during RAIT in a dog with functional thyroid carcinoma, followed by successful long-term control. The outcome suggests that prompt recognition and stabilization of thyrotoxicosis-related crises did not preclude the safe and effective use of RAIT. More broadly, this case indicates the importance of individualized RAIT protocols that incorporate scintigraphic assessment, dose adjustment, and consideration of comorbidities to optimize clinical outcomes in dogs with thyroid carcinoma presenting with thyrotoxicosis.

## Figures and Tables

**Table 1:** Serial hematological, biochemical, and hormonal findings during the treatment course.

Parameters	Before 1st RAIT	3 Months After 1st RAIT	1 Month After 2nd RAIT	8 Months After 2nd RAIT
tT4 [1.0–4.0 ug/dL]	12.1	11.7	3.01	1.75
TSH [0.05–0.42 ng/mL]	<0.030	0.076	<0.030	0.052
WBC [5.05–16.76 × 10^3^/μL]	19.79	17.70	11.72	10.19
PCV [37.3–61.7%]	49.4	46.8	47.2	39.0
PLT [148–484 × 10^3^/μL]	421	404	353	357
BUN [7–25 mg/dL]	27.4	30.5	28.7	31.4
Crea [0.5–1.5 mg/dL]	0.9	1.0	1.3	0.98
ALT [21–102 IU/L]	58	31	32	19
ALP [29–97 IU/L]	650	505	470	302
Lac [0.5–2.5 mmol/L]	8.55	3.65	N/A	N/A
CRP [0–10 mg/L]	17.53	20.91	8.66	5.06

Abbreviations: RAIT, radioactive iodine therapy; tT4, total thyroxine; TSH, thyroid-stimulating hormone; WBC, white blood cell count; PCV, packed cell volume; PLT, platelet count; BUN, blood urea nitrogen; Crea, creatinine; ALT, alanine aminotransferase; ALP, alkaline phosphatase; Lac, blood lactate; CRP, C-reactive protein; N/A, not assessed.

**Table 2:** Canine-adapted Burch–Wartofsky Point Scale for thyroid storm ^a^.

Diagnostic Parameters	Criteria	Points	Case Score
1. Thermoregulatory dysfunction: body temperature (°C) ^b^	39.5–39.9	5	+5
	40.0–40.8	10	
	40.9–41.7	20	
	≥41.8	30	
2. CNS dysfunction	Absent	0	+10
	Mild (agitation)	10	
	Moderate (delirium, psychosis, and extreme lethargy)	20	
	Severe (seizure and coma)	30	
3. Gastrointestinal-hepatic dysfunction	Absent	0	+10
	Moderate (diarrhea, nausea/vomiting, and abdominal pain)	10	
	Severe (unexplained jaundice)	20	
4. Cardiovascular dysfunction: heart rate (bpm) ^c^	140–179	5	+25
	180–199	10	
	200–239	15	
	≥240	25	
5. Cardiovascular dysfunction: congestive heart failure	Absent	0	+0
	Mild (pedal edema)	5	
	Moderate (bibasilar rales)	10	
	Severe (pulmonary edema)	15	
6. Cardiovascular dysfunction: atrial fibrillation	Absent	0	+0
	Present	10	
7. Precipitant history	Absent	0	+10
	Present	10	
Total score			60

^a^ Table reproduced from Burch & Wartofsky (1993) [2] with canine-specific modifications. A score of 45 or greater is highly suggestive of thyroid storm; a score of 25 to 44 is suggestive of impending storm, and a score below 25 is unlikely to represent thyroid storm. ^b^ Adjusted for canine-specific normal body temperature and fever severity thresholds aligned with VCOG-CTCAE v2 definitions [20]. ^c^ Adjusted for canine-specific normal heart rate and tachycardia severity thresholds aligned with VCOG-CTCAE v2 definitions [20]. CNS, central nervous system; VCOG-CTCAE v2, Veterinary Cooperative Oncology Group—Common Terminology Criteria for Adverse Events.

## Data Availability

The original contributions presented in this study are included in the article. Further inquiries can be directed to the corresponding author.
